# The GABAergic Hypothesis for Cognitive Disabilities in Down Syndrome

**DOI:** 10.3389/fncel.2017.00054

**Published:** 2017-03-07

**Authors:** Andrea Contestabile, Salvatore Magara, Laura Cancedda

**Affiliations:** ^1^Department of Neuroscience and Brain Technologies, Istituto Italiano di Tecnologia (IIT)Genova, Italy; ^2^Dulbecco Telethon InstituteGenova, Italy

**Keywords:** GABA, down syndrome, cognitive impairment, gaba receptors, chloride homeostasis

## Abstract

Down syndrome (DS) is a genetic disorder caused by the presence of a third copy of chromosome 21. DS affects multiple organs, but it invariably results in altered brain development and diverse degrees of intellectual disability. A large body of evidence has shown that synaptic deficits and memory impairment are largely determined by altered GABAergic signaling in trisomic mouse models of DS. These alterations arise during brain development while extending into adulthood, and include genesis of GABAergic neurons, variation of the inhibitory drive and modifications in the control of neural-network excitability. Accordingly, different pharmacological interventions targeting GABAergic signaling have proven promising preclinical approaches to rescue cognitive impairment in DS mouse models. In this review, we will discuss recent data regarding the complex scenario of GABAergic dysfunctions in the trisomic brain of DS mice and patients, and we will evaluate the state of current clinical research targeting GABAergic signaling in individuals with DS.

## Introduction

Down syndrome (DS) or trisomy 21 is the leading cause of genetically-defined intellectual disability and congenital birth defects. DS is characterized by many phenotypical features affecting almost all body systems, including developmental defects and growth delay (Nadel, 2003; Antonarakis and Epstein, 2006). In particular, brains of individuals with DS show decreased volume and reduced neuronal density in diverse brain areas (e.g., cortex, hippocampus and cerebellum; Sylvester, 1983; Coyle et al., 1986; Aylward et al., 1997, 1999; Shapiro, 2001). These alterations originate early during development (Schmidt-Sidor et al., 1990; Winter et al., 2000; Pinter et al., 2001; Larsen et al., 2008) and are possibly due to defective neuronal precursor proliferation during gestation (Contestabile et al., 2007; Guidi et al., 2008). Accordingly, the development of individuals with DS is characterized by delayed cognitive progress in infancy and childhood, leading to mild-to-moderate mental retardation with an Intelligence Quotient (IQ) ranging from 30 to 70 (Vicari et al., 2000, 2005; Pennington et al., 2003; Vicari, 2004). This scenario is additionally worsened during adulthood by further loss of cognitive abilities and the development of Alzheimer’s disease (AD) by the fourth decade of life (Wisniewski et al., 1985; Mann and Esiri, 1989; Leverenz and Raskind, 1998; Teipel and Hampel, 2006).

Although cognitive impairment is the most common and severe feature of DS, other neurological and psychiatric manifestations of the disease highly impinge on the quality of life of individuals with DS and their families. In particular, the DS population shows increased frequency of anxiety (Vicari et al., 2013), clinically relevant sleep disturbance (Carter et al., 2009; Breslin et al., 2011; Angriman et al., 2015; Edgin et al., 2015; Konstantinopoulou et al., 2016; Maris et al., 2016), and hyperactivity or movement disorders (Pueschel et al., 1991; Haw et al., 1996). Finally, DS patients demonstrate increased incidence of epileptic episodes with seizure onset mainly concentrated during early life and aging (Stafstrom et al., 1991; Lott and Dierssen, 2010; Robertson et al., 2015).

The identification of possible mechanisms leading to cognitive impairment in DS has been largely conducted thanks to the analysis of different DS genetic mouse models (Dierssen, 2012). Interestingly, compelling studies in these animals indicate that altered signaling of the neurotransmitter GABA is one of the main determinants in reducing cognitive and memory functions. In particular, GABAergic dysfunctions impair synaptic plasticity and learning and memory in DS by altering optimal excitatory/inhibitory synaptic balance (Kleschevnikov et al., 2004, 2012a,b; Costa and Grybko, 2005; Fernandez et al., 2007; Deidda et al., 2014). In this review article, we will survey available data on the alteration of GABAergic signaling in DS trisomic animal models and individuals with DS. Additionally, since no effective pharmacological treatment for ameliorating cognitive deficits in DS has been found yet, we will also evaluate the current status of pre-clinical and clinical trials with GABAergic drugs in DS.

## GABA Signaling in The Brain

GABA is the main inhibitory neurotransmitter in the adult and healthy brain and it acts through ionotropic and metabotropic receptors (GABA_A_Rs and GABA_B_Rs, respectively).

### GABA_A_ Receptors

GABA_A_Rs are chloride-permeable ion channels. In the adult brain, the chloride gradient across the neuronal cell membrane is sustained by the exporter K-Cl cotransporter (KCC2), which maintains low intracellular chloride concentration ([Cl^−^]_i_). Thus, GABA_A_R opening generates an influx of negative chloride ions that hyperpolarizes the cell membrane potential and inhibits neuronal activity. Moreover, opening of GABA_A_R ion channels may also shunt concurrent excitatory currents (e.g., driven by the neurotransmitter glutamate), thus preventing them from bringing the membrane potential to the action potential threshold. Indeed, GABA_A_R opening short-circuits depolarizing synaptic currents by locally reducing the input resistance (Ben-Ari, 2002; Jonas and Buzsaki, 2007; Silver, 2010; Khazipov et al., 2015). Thus, adult GABA_A_-mediated transmission is physiologically hyperpolarizing and inhibitory. Conversely, in early neurodevelopment, KCC2 expression is low, and associated with high expression of the chloride importer Na-K-Cl cotransporter (NKCC1), which generates high [Cl^−^]_i_. In this condition, the chloride flux through GABA_A_Rs is outward and depolarizes the cell membrane potential (Cherubini et al., 1990; Rivera et al., 1999; Ben-Ari, 2002; Fiumelli et al., 2005). Although GABA_A_R-mediated depolarizing postsynaptic potentials (PSPs) will be in most cases not sufficient to reach the threshold for action-potential generation, they will be able to activate voltage-gated calcium channels (VGCCs), remove the voltage-dependent magnesium block from NMDA receptors (Leinekugel et al., 1995, 1997; Ben-Ari, 2002), and possibly add up to concurrent excitatory inputs (Gulledge and Stuart, 2003). Consequently, even an initially mild depolarizing effect of GABA can contribute to a more pronounced excitation and eventually reach the threshold for action potential firing. Thus, GABA_A_-mediated transmission is generally depolarizing and possibly excitatory in the immature brain, although depolarizing GABA_A_ currents may still be inhibitory by shunting concurrent excitatory inputs. Notably, a depolarizing (rather than hyperpolarizing) GABA_A_R response may also occur in mature neurons depending on timing and location of GABA_A_Rs, upon relatively small difference in [Cl^−^]_i_ at different cell compartments_,_ more hyperpolarized resting membrane potentials, sustained activity or even pathological conditions (Lamsa and Taira, 2003; Khirug et al., 2008; Chiang et al., 2012; Cellot and Cherubini, 2014). Indeed, as the reversal potential for GABA_A_R-mediated chloride currents (*E*_Cl_) sits very near to the resting membrane potential (*V*_REST_), relatively small changes in [Cl^−^]_i_ are sufficient to change the polarity of GABAergic responses. In particular, if *E*_Cl_ is more negative than *V*_REST_, the chloride flow will be inward (hyperpolarizing). Instead, if *E*_Cl_ is less negative than *V*_REST_, the chloride flow will be in the opposite direction, generating depolarizing PSPs.

GABA_A_Rs are composed of five subunits, and the many subunit variants identified in mammals (six α, three β, three γ, one δ, three ρ, one ε, one π and one θ) confer different pharmacological and kinetic properties to the receptor (Olsen and Sieghart, 2008; Fritschy and Panzanelli, 2014). In the CNS, the largest population of GABA_A_Rs is composed of 2 α, 2 β and 1 γ subunits, but isoform expression and receptor composition change with subcellular localization, brain area, and development, thus assuring receptor properties set to fulfil specific neuronal needs. For example, the α_1–3_, β_2–3_ and γ_2_ isoforms are the most represented in the adult brain, constituting synaptic GABA_A_Rs. In particular, thanks to their fast kinetics with rapid onset and desensitization, they generate phasic currents following GABA release from presynaptic vesicles with spatial and temporal accuracy. Conversely, α_5_- or δ-containing GABA_A_Rs are mainly extra-synaptic. The presence of the α_5_ or δ subunits confers high GABA affinity and slow desensitization kinetics to GABA_A_Rs. Thus, α_5_ or δ–containing receptors are able to detect ambient GABA spilled out of the synaptic cleft and generate long-lasting (tonic/extrasynaptic) currents (Böhme et al., 2004; Caraiscos et al., 2004; Farrant and Nusser, 2005; Zheleznova et al., 2009).

Phasic vs. tonic GABA_A_-mediated currents provide for different regulation of neuronal physiology. Phasic GABA transmission plays a relevant role in synaptic-input integration. Indeed, fast inhibition acts as coincidence detector of excitatory inputs by feed-forward inhibition: this mechanism ensures that a second input desynchronized with a first input is canceled out; thus, excitatory inputs sum up only when they are perfectly synchronized (Pouille and Scanziani, 2001). A similar although opposite integration occurs when GABA is depolarizing. GABA_A_R opening may generate small excitatory potentials that last after the channel closure and sum up with concurrent excitatory inputs, thus facilitating the achievement of action potential threshold (Gulledge and Stuart, 2003). While phasic inhibition participates in synaptic integration, tonic inhibition generates relatively persistent changes of input conductance, thus rightward shifting the input-output relationship. Therefore, tonic inhibition reduces the magnitude, duration and propagation length of excitatory PSPs, limiting their temporal/spatial summation possibilities and inhibiting neuronal excitability. Such effect is mainly mediated by shunting which is particularly relevant for the inhibitory action of tonic GABA transmission and less for the phasic inhibition, given the longer opening of tonic GABA_A_Rs (Ben-Ari, 2002; Farrant and Nusser, 2005; Jonas and Buzsaki, 2007; Silver, 2010; Khazipov et al., 2015).

Integration processes of excitatory inputs depend from PSPs generated from GABA_A_Rs, shunting inhibition, and also from the localization of GABA_A_Rs. For example, on the dendrites of CA1 pyramidal neurons, GABAergic synapses are mostly located on the dendritic shaft and on spines (Megías et al., 2001), thus participating to synaptic integration of two different sets of inputs: the ones already partially processed along the way from their synaptic origin to the dendritic shaft, and the ones originated in neighborhood synapses in the same spine, respectively. Notably, different cellular compartments show different [Cl^−^]_i_ that critically determine GABA_A_R participation to input integration in a compartment-specific fashion. Indeed, [Cl^−^]_i_ is maximum in the axon and axonal initial segment, lower in the soma, and it continues to decrease gradually along dendrites (Szabadics et al., 2006; Khirug et al., 2008; Waseem et al., 2010). In summary, phasic inhibition plays as a coincidence detector and undergoes to tight spatial and temporal integration with excitatory inputs in a compartment-specific manner; tonic inhibition sets the neuronal excitability background, by affecting excitatory input-output relation.

### GABA_B_ Receptors

GABA_B_Rs are G_i/o_-protein-coupled receptors (GPCRs) mostly localized extra-synaptically. Their high affinity for GABA ensures that ambient GABA spilled out of synapses can activate GABA_B_Rs despite of their distance. In particular, the GABA_B_R Gα_i/o_ subunit can lead to adenylate cyclase inhibition and consequent reduction of cAMP levels and PKA pathway activity (Bettler et al., 2004; Padgett and Slesinger, 2010); Gβγ_i/o_ subunit, instead, inhibits voltage-gated calcium channels and opens G protein-coupled inwardly-rectifying potassium channels (GIRK/Kir3), tetramers formed by different compositions of GIRK1–4 subunits (Koyrakh et al., 2005). In particular, presynaptic GABA_B_Rs reduce vesicle release by inhibition of the voltage-gated calcium channels and by a calcium independent mechanism (Rost et al., 2011), whereas GABA_B_R subunits found on dendrite and spine necks regulate neuronal excitability by coupling to the GIRK channels (Nicoll, 2004; Koyrakh et al., 2005). Interestingly, all GIRK subunits virtually form protein complexes with GABA_B_R subunits in heterologous systems (David et al., 2006; Fowler et al., 2007; Ciruela et al., 2010). Nevertheless, GABA_B_R-triggered potassium currents seem to be mostly mediated by GIRK2-containing homo- and hetero-tetramers (Koyrakh et al., 2005). GIRK channel opening elicits an outward hyperpolarizing K^+^ current and a decrease in the input resistance (Lüscher et al., 1997; Koyrakh et al., 2005). Thus, through GIRK channels, GABA_B_Rs inhibit neuronal excitability by shunting excitatory currents, hyperpolarizing the membrane with slow inhibitory PSPs, and contributing in maintaining *V*_REST_ (Lüscher et al., 1997; Koyrakh et al., 2005; Gassmann and Bettler, 2012). These effects also prevent action potential back-propagation in dendrites (Leung and Peloquin, 2006). Finally, through inhibition of postsynaptic voltage-gated calcium channels, GABA_B_R activation also prevents dendritic calcium spikes (Chalifoux and Carter, 2011).

### GABA_A_ and GABA_B_ Receptor Functions Across Neurodevelopment and Adulthood

Given the variety of potential signaling dynamics by GABA_A_ and GABA_B_ receptors, it is not surprising that GABA may play a relevant role in different processes during healthy neurodevelopment and adulthood, as well as under pathological conditions. In particular, depolarizing and possibly excitatory GABA_A_-mediated transmission in early life plays key roles in promoting neurodevelopment. α_5_- or δ-containing extrasynaptic GABA_A_Rs are predominant at this stage when synapses are not yet formed and ambient GABA released from growth cones and astrocytes is detected by high affinity subunits (Cellot and Cherubini, 2013). Depolarizing tonic currents facilitate spiking, thus increasing the probability of firing coincidence from different cells and therefore of synaptic wiring important for synaptogenesis. Moreover, these tonic currents drive neuronal migration and maturation, axon growth, and synaptic plasticity (Ben-Ari et al., 2007; Wang and Kriegstein, 2009; Kilb et al., 2013; Luhmann et al., 2015). Also GABA_B_Rs regulate neuronal migration, maturation of pyramidal neurons, synapse formation and circuit development (Fiorentino et al., 2009; Gaiarsa and Porcher, 2013). Notably, GABA_B_R activation is not able to elicit GIRK-mediated (hyperpolarizing) currents in early neurodevelopment, although maintaining the pre-synaptic inhibitory function on neurotransmitter release. Indeed, coupling of postsynaptic GABA_B_Rs to Kir channels is delayed during development (Fukuda et al., 1993; Gaiarsa et al., 1995; McLean et al., 1996).

Finally, in neurodevelopment, both GABA_A_- and GABA_B_-mediated transmission control synaptic plasticity. Indeed, GABA_A_ receptors interfere both with the opening and the closure of the visual cortex critical period, i.e., the time window when brain plasticity can be evoked by environmental stimuli and experimental paradigms in slices and *in vivo* (i.e., long-term potentiation (LTP) and induction and monocular deprivation, respectively; Levelt and Hübener, 2012). Eventually, in the adult brain, GABA_A_Rs usually exert a negative regulation on hippocampal plasticity and cognition. Indeed, GABA_A_R-mediated inhibition suppresses LTP both *in vitro* and *in vivo* (Wigström and Gustafsson, 1986; Grover and Yan, 1999; Matsuyama et al., 2008), and benzodiazepines (positive regulators of GABA_A_Rs) or GABA_A_R activation impairs memory (Roth et al., 1984; Zarrindast et al., 2002; Raccuglia and Mueller, 2013). While LTP is enhanced by GABA_A_R blockade (Wigström and Gustafsson, 1986), long term depression (LTD) is facilitated by GABA_A_R activation *in vitro* (Steele and Mauk, 1999), thus suggesting that GABA_A_-mediated inhibition balances the ratio between LTP and LTD. On the other hand, GABA_B_R-mediated membrane hyperpolarization, inhibition of voltage-gated calcium channels and of back-propagating spikes, as well as reduction of the cAMP/PKA generally contribute to prevent synaptic plasticity. Indeed, GABA_B_Rs generally suppress LTP and memory performance, however a dual role in plasticity regulation is mediated by auto- and hetero-GABA_B_Rs (Davies et al., 1991; Stäubli et al., 1999). Finally, GABAergic regulation of brain plasticity also involves adult neurogenesis. Indeed, tonic depolarizing GABA_A_ responses by GABAergic Parvalbumin interneurons negatively regulate adult neurogenesis in the dentate gyrus (DG) of the hippocampus (Song et al., 2012; Pontes et al., 2013; Pallotto and Deprez, 2014).

Phasic GABA_A_-mediated transmission also ensures simultaneous and temporally limited inhibition, able to synchronize network activity and generate network oscillations, according to both computational models and *in vitro* slice experiments from juvenile (P14–27) and adult mice (Wang and Buzsáki, 1996; Mann and Mody, 2010). Moreover, GABA_B_Rs synchronize hippocampal network activity at low oscillation frequency (Scanziani, 2000; Kohl and Paulsen, 2010) and are activated during cortical up-states, contributing to their termination (Mann et al., 2009). Finally, GABA also exerts key roles in pathological conditions such as a number of neurodevelopmental disorders (Ramamoorthi and Lin, 2011; Deidda et al., 2014), epilepsy (Kaila et al., 2014a), anxiety (Nuss, 2015), and neurodegenerative diseases (e.g., AD; Li et al., 2016).

## Mouse Models of DS

Most of the current knowledge regarding alterations of the GABAergic signaling in the DS brain has come from the study of mouse models of DS. According to a recent study utilizing the Vertebrate Genome Annotation (VEGA) database^1^, the human chromosome 21 (Hsa21) contains a total of 222 protein-coding genes (of which 218 map to the long arm 21q), including two large clusters of 49 keratin-associated proteins (KRTAPs; Gupta et al., 2016). The mouse genes orthologue of those mapping to the long arm of Hsa21 are distributed on three syntenic regions present on mouse chromosomes 10, 16 and 17. In particular, the distal portion of mouse chromosome 16 (Mmu16) encompasses a large (~28 Mb) region that contains ~55% of Hsa21 orthologous protein-coding genes (Antonarakis et al., 2004; Gupta et al., 2016). Therefore, many of the available DS mouse models have been created by genetic manipulation of this Mmu16 region. Specifically, the Ts65Dn mouse (Reeves et al., 1995) is the most widely used murine model of DS and carries an extra translocation chromosome composed of the Mmu16 syntenic region fused to the centromeric portion of Mmu17. This freely-segregating extra chromosome contains 90 non-KRTAP, Hsa21 protein-coding orthologues, plus 35 protein-coding genes (deriving from Mmu17) that are not triplicated in DS (Duchon et al., 2011; Gupta et al., 2016). Additional DS mouse models carrying a smaller triplication of the Mmu16 syntenic region are the Ts1Cje and the Ts1Rhr. Ts1Cje mice are characterized by the genomic duplication of a Mmu16 segment containing 71 Hsa21 protein-coding orthologues and translocated to the distal portion of Mmu12 (Sago et al., 1998; Gupta et al., 2016). However, the translocation resulted in the deletion of seven genes in the most telomeric segment of Mmu12 (Duchon et al., 2011). Ts1Rhr mice (Olson et al., 2004) were generated by Cre/lox chromosome engineering and carry a tandem duplication of an even smaller Mmu16 region comprising 29 Hsa21 protein-coding orthologues from the so-called “DS critical region” (DSCR; Delabar et al., 1993; Korenberg et al., 1994).

The vast majority of the studies on DS-related cognitive and electrophysiological abnormalities have been performed on the Ts65Dn mouse. Indeed, although the Ts65Dn model still presents issues from a genetic point of view (Gardiner et al., 2003), it recapitulates many of the phenotypic features of the human syndrome (Dierssen, 2012; Rueda et al., 2012), and it is currently the only mouse model used for preclinical identification of pharmacological interventions targeting DS cognitive impairment (Gardiner, 2014). Moreover, phenotypic comparison of different DS mouse models has suggested that the genes triplicated in the Ts65Dn mouse are major responsible for DS-related cognitive abnormalities (Rueda et al., 2012). In particular, Ts65Dn mice show severe behavioral deficits in different learning and memory tasks, including fear conditioning, T-maze spontaneous alternation, Morris water maze and object recognition tests (Reeves et al., 1995; Costa et al., 2007; Fernandez et al., 2007; Contestabile et al., 2013), and electrophysiological alterations in both synaptic transmission (Kleschevnikov et al., 2004, 2012b; Best et al., 2007, 2012; Hanson et al., 2007; Mitra et al., 2012) and hippocampal synaptic plasticity (Siarey et al., 1997, 1999; Kleschevnikov et al., 2004; Costa and Grybko, 2005; Contestabile et al., 2013). In addition, Ts65Dn mice display alterations in dendritic spine morphology (Belichenko et al., 2004; Guidi et al., 2013) and impaired neurogenesis both in the developing brain (Baxter et al., 2000; Roper et al., 2006; Chakrabarti et al., 2007, 2010; Contestabile et al., 2007, 2009) and in neurogenic niches of the adult brain (Clark et al., 2006; Bianchi et al., 2010a; Contestabile et al., 2013). Additionally, Ts65Dn mice are not spontaneously epileptic, but show increased seizures incidence in some experimental epilepsy paradigms (Cortez et al., 2009; Westmark et al., 2010; Joshi et al., 2016). Finally, similarly to DS patients, Ts65Dn mice also exhibit some sleep alterations and hyperactivity in locomotor behavior (Escorihuela et al., 1995; Reeves et al., 1995; Sago et al., 2000; Colas et al., 2008).

Although Ts1Cje and Ts1Rhr mice show some DS-related phenotypes, the extent of these features is somewhat milder and with some distinct characteristics (Rueda et al., 2012). In particular, Ts1Cje mice displayed deficits in synaptic plasticity and behavior in the T-maze test comparable to Ts65Dn mice, whereas memory performances were less severely affected in the Morris water maze and substantially spared in the novel object recognition (Sago et al., 1998; Siarey et al., 2005; Belichenko et al., 2007; Fernandez and Garner, 2007). On the other hand, Ts1Rhr mice showed impaired synaptic plasticity in the hippocampal DG, but unaffected plasticity in the CA1 hippocampal region. At the behavior level, deficits were detected in the *T*-maze and novel object recognition tests, but not in the Morris water maze test (Olson et al., 2007; Belichenko P. N. et al., 2009). Finally, Ts1Cje mice were found hypoactive (Sago et al., 1998), and Ts1Rhr mice mostly not different compared to WT animals for locomotor behavior (Belichenko P. N. et al., 2009).

In more recent years, systematic application of Cre/lox-mediated chromosome engineering has permitted the creation of three new murine lines (Dp10, Dp16 and Dp17) individually trisomic (through a tandem duplication on the corresponding chromosome) for the three complete syntenic regions on Mmu10, 16 and 17, respectively (Li et al., 2007; Yu et al., 2010a, b). Nevertheless, although these mouse lines represent ideal models in terms of genetic triplication, they lack the extra freely-segregating chromosome that is found in most individuals with DS and in Ts65Dn mice. In fact, the presence of an extra unpaired chromosome could also have a role *per se* in the phenotypic consequences of trisomy by impacting on global gene expression and/or chromatin structure (Reinholdt et al., 2009; Dierssen, 2012). On the other hand, mice fully-trisomic for all Hsa21 orthologue genes can also be created by successively crossing the Dp10, Dp16 and Dp17 lines, although decreased viability and poor breading have limited the experimental studies on these triple trisomic mice (Yu et al., 2010a; Belichenko et al., 2015). Moreover, the Dp10, Dp16 and Dp17 mice have also permitted the dissection of the relative contribution of the different triplicated regions to the diverse disease phenotypes. Interestingly, while Dp16 and triple trisomic mice (Dp10/Dp16/Dp17) show behavioral and synaptic plasticity deficits comparable to the ones found in Ts65Dn mice (Yu et al., 2010a,b; Belichenko et al., 2015), and the single trisomic mice Dp10 as well as Dp17 show normal (or even enhanced) performances (Yu et al., 2010b), highlighting the importance of the Mmu16 syntenic region in DS-related phenotype.

## GABA Signaling in DS Mouse Models

### GABA_A_ Signaling and Trisomy

**Figure 1:**
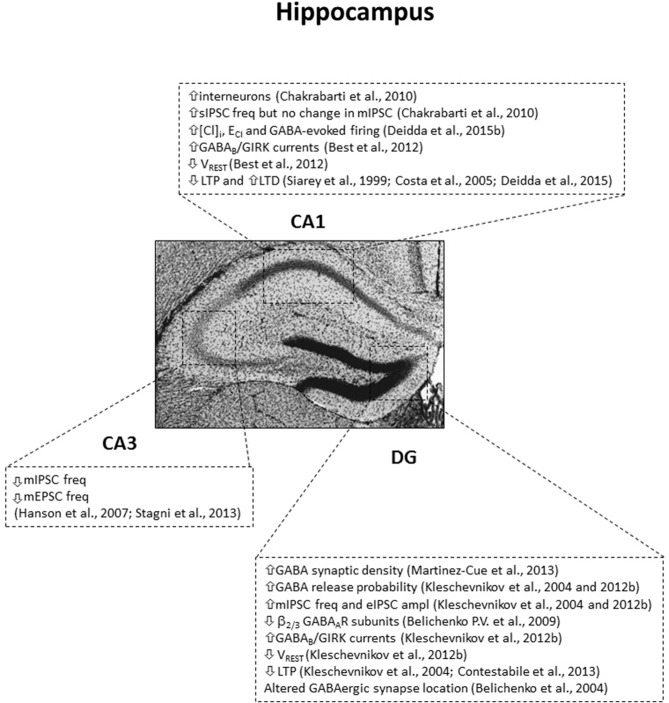
**Subregion-specific GABA-related dysfunctions in the hippocampus of the Ts65Dn mouse model.** IPSC, inhibitory postsynaptic currents; sIPSC, spontaneous IPSC; mIPSC, miniature IPSC; eIPSC, evoked IPSC; freq, frequency; ampl, amplitude; mEPSC, miniature excitatory postsynaptic currents; [Cl]_I_, intracellular chloride concentration; *E*_Cl_, reversal potential of GABA_A_R-mediated Cl currents; *V*_REST_, resting membrane potential. Arrows indicate increases or decreases of the reported measures.

A first main finding regarding GABA-related phenotypes in DS came from the observation that the number of GABAergic interneurons is increased in both the cortex and the hippocampus of Ts65Dn mice (Chakrabarti et al., 2010; Pérez-Cremades et al., 2010; Hernández et al., 2012; Hernández-González et al., 2015). Specifically, this increase arises from amplified neurogenesis during embryonic development of neural progenitor cells in the medial ganglionic eminence (MGE, where most interneurons originate during development; Marín and Müller, 2014), and it is more prominent for Parvalbumin and Somatostatin-positive GABAergic interneurons (Chakrabarti et al., 2010). In line with the increase in GABAergic interneurons, the same authors reported also an increase in spontaneous GABAergic postsynaptic events in CA1 pyramidal neurons. Nevertheless, further functional analysis of GABAergic transmission by electrophysiology did not find evidence for alterations in the frequency of miniature inhibitory postsynaptic currents (mIPSC, which represent activity-independent quantal release of GABA), release probability at GABAergic synapses and evoked GABA_A_ transmission in the hippocampal CA1 region of adult Ts65Dn mice (Chakrabarti et al., 2010; Best et al., 2012). Therefore, the increase of spontaneous GABAergic events observed in Ts65Dn mice may be due to a general enhancement in interneuron excitability rather than a specific increase in the number of GABAergic synapses. Indeed, further electron microscopy studies in the temporal cortex and hippocampus of adult Ts65Dn mice found the density of symmetric synapses (putative GABAergic) to be unaffected (Kurt et al., 2000, 2004; Belichenko P. V. et al., 2009). Similarly, immunohistochemical evaluation of GABAergic terminals confirmed comparable density between WT and Ts65Dn mice in the DG, even if the distribution of GABAergic synapses appeared altered, with a selective redistribution of GABAergic synapses from the dendrite shaft and the spine heads to the spine neck of trisomic neurons (Belichenko et al., 2004; Belichenko P. V. et al., 2009; Kleschevnikov et al., 2012b). Such selective retribution of GABAergic synapses to the spine neck was later also confirmed in the DG of Ts1Cje mice (Belichenko et al., 2007). The altered location of GABAergic synapses in DS mice may impair synaptic input integration. Indeed, GABAergic contacts on the spine neck may account for integration of spine-converging inputs, in replacement of contacts on the spine head and on the dendritic shaft that integrate inputs at local synaptic and dendritic levels, respectively. Anyhow, the increased number of GABAergic neurons seems not to be directly associated with increased synaptic contacts in the DS brain. The reason is not fully understood, but GABAergic synaptic density may normalize in adulthood for compensatory mechanisms, after defective brain development. Indeed, differently than in adult Ts65Dn mice, both mIPSC frequency and evoked GABA_A_ transmission (but not release probability) were found larger in the CA1 region of 2 weeks old Ts65Dn mice (Mitra et al., 2012). Further support to the compensation hypothesis is the observation that an increased GABAergic synaptic density was in fact found specifically in the inner molecular layer and granular layer of the hippocampal DG of adult Ts65Dn mice (Martínez-Cué et al., 2013; García-Cerro et al., 2014; Mojabi et al., 2016). Indeed, this brain area is highly innervated by GABAergic fibers, and one could expect that developmental compensatory mechanisms may be more difficult to put in place. On the other hand, all these seemingly conflicting results may simply indicate that GABA_A_-mediated dysfunctions are not uniform in all areas of the trisomic brain and/or may also be simply due to age-related changes. Indeed, mIPSC frequency and evoked GABAergic transmission were found increased in the DG of adult Ts65Dn mice, but with this alteration primarily attributed to increased release probability at GABAergic terminals, rather than increased synapse number (Kleschevnikov et al., 2004, 2012b), indicating possible sub-regional differences in the density of GABAergic synapses and its functional consequences (see Figure 1). Additionally, mIPSC frequency was found decreased (rather than increased) in the hippocampal CA3 region of Ts65Dn mice (Hanson et al., 2007; Stagni et al., 2013). Finally, outside the hippocampus, increased excitability, enhanced release probability and decreased tonic GABA inhibition were found in cerebellar granule cells of Ts65Dn mice (Usowicz and Garden, 2012; Das et al., 2013; Szemes et al., 2013).

Phasic and tonic GABA transmission is necessary to synchronize neuronal firing and maintain physiological network oscillation. Interestingly, gamma oscillation power is reduced in Ts65Dn hippocampal slices, while duration and frequency of up-states are reduced in Ts65Dn layer 4 of somatosensory cortex slices (Hanson et al., 2013; Cramer et al., 2015).

Regarding the expression of the different GABA receptors, no statistical difference was found in the hippocampus of Ts65Dn mice compared to WT for *α*_1_, *α*_2_, *α*_3_, *α*_5_, *γ*_2_ subunits of GABA_A_R (Belichenko P. V. et al., 2009; Best et al., 2012; Kleschevnikov et al., 2012b). However, decreased immunostaining for β_2/3_ subunits of GABA_A_R have been reported in the DG of 3 month-old Ts65Dn mice, but not at later time points, suggesting possible age-related changes (Belichenko P. V. et al., 2009). Moreover, the binding of the selective α_5_ subtype radio-ligand [^3^H]RO0154513 was unchanged *in vivo* in Ts65Dn mice compared to WT, suggesting similar expression of GABA_A_Rs containing the α_5_ subunit (Martínez-Cué et al., 2013).

As detailed above, GABA_A_R-mediated transmission undergoes a developmental switch from depolarizing (excitatory) to hyperpolarizing (inhibitory) during neuronal maturation and it can shift between inhibition and excitation under some circumstances and/or in particular cellular compartments (Andersen et al., 1980; Staley et al., 1995; Gulledge and Stuart, 2003; Viitanen et al., 2010; Ruusuvuori et al., 2013). Consequently, the complex relationship of ionic movements determined by GABA_A_R signaling and chloride transporters must be highly regulated to avoid deleterious consequences on neuronal physiology (Deidda et al., 2014). In this regard, we have recently found that NKCC1 is upregulated in the brain of both Ts65Dn mice and individuals with DS (Deidda et al., 2015b). Accordingly, we found that [Cl^−^]_i_ was increased in CA1 pyramidal neurons from Ts65Dn mice and *E*_Cl_ was depolarized by 7.7 mV compared to WT neurons (Deidda et al., 2015b). As a result, *E*_Cl_ was less negative than *V*_REST_ in trisomic neurons, thus predictive of outward Cl^−^ depolarizing currents upon GABA_A_R activation. Accordingly, the mean firing frequency of individual CA1 neurons was increased by exogenous application of GABA in trisomic hippocampal slices, whereas it was decreased by blockade of GABA_A_R-mediated transmission with bicuculline, the opposite of what physiologically observed in WT neurons (Deidda et al., 2015b). Therefore, despite the complex dual excitatory and shunting/inhibitory effect of GABA following *E*_Cl_ depolarization, GABA_A_ signaling was mainly excitatory in Ts65Dn neurons in our experimental conditions of prolonged bath-application of the drugs in acute brain slices. In this regard, the contribution of evoked, synaptically released or tonic GABA signaling to the depolarizing effect of GABA_A_Rs has not been studied yet in Ts65Dn slices. Similarly, experiments are needed to evaluate *in vivo* the strength and direction of GABA_A_ transmission in DS. Moreover, little is known about the contribution of GABAergic signaling on network dynamics in trisomic neurons. However, one calcium imaging study on hippocampal cultures showed decreased response to bicuculline application on network burst-amplitude and duration in Ts65Dn mice, as well as decreased network bursts upon GABA application in both WT and Ts65Dn culture (Stern et al., 2015).

Despite of whether GABA_A_R-induced depolarization may reach the threshold for neuronal excitation or not, GABA_A_R-driven depolarization may still work in favor of excitation more efficiently than GABA_A_R-driven shunting would work in favor of inhibition. Indeed, in Ts65Dn neurons the shunting effect of GABA_A_ opening may be very mild on dendritic signal processing, since most synapses are located on the spine neck. Instead, GABA_A_ depolarizing PSPs may generate in the spine neck for the depolarized *E*_Cl_ and propagate to spine heads and dendritic shaft, consequently affecting NMDA receptor and voltage-gated channel openings, and adding up to concurrent excitatory PSPs. Notably, the concomitant hyperpolarized shift of *V*_REST_ in Ts65Dn neurons, due to GIRK2 triplication (Best et al., 2012), may also contribute to the depolarizing action of GABA. Anyhow, pharmacological inhibition of NKCC1 transport activity with the specific antagonist Bumetanide completely restored the hyperpolarizing and inhibitory action of GABA in Ts65Dn neurons (Deidda et al., 2015b).

### GABA_B_ Signaling and Trisomy

Another direct link between DS genetic triplication and GABA signaling came from the discovery that the KCNJ6 gene, which encodes the subunit 2 of the GIRK channel (GIRK2/Kir3.2), maps to Hsa21 (Ohira et al., 1997; Hattori et al., 2000). The presence of an extra KCNJ6 copy in Ts65Dn mice leads to overexpression of GIRK2 mRNA and protein in hippocampus, cortex and midbrain (Harashima et al., 2006). Interestingly, also the GIRK1 protein (which is not triplicated in DS) is overexpressed in Ts65Dn brains, with normal levels of mRNA. Thus, the increased GIRK1 protein expression is most likely due to enhanced hetero-dimerization with GIRK2 subunit (which drives GIRK subunit trafficking) and downstream decreased protein turnover (Harashima et al., 2006). As a consequence, GABA_B_/GIRK currents are increased in cultured primary hippocampal neurons (Best et al., 2007), as well as in CA1 pyramidal neurons and in DG granule cells from acute slices of the Ts65Dn hippocampus (Best et al., 2012; Kleschevnikov et al., 2012b). This increase of GABA_B_/GIRK signaling in Ts65Dn neurons could strongly affect neuronal excitability and plasticity by enhancement of GIRK-mediated shunting inhibition, reduction of excitatory PSPs, back-propagating action potentials, and change of neuronal passive properties (Lüscher et al., 1997; Koyrakh et al., 2005). Anyhow, genetically restoring GIRK2 gene dosage to disomy in Ts65Dn mice (by crossing to GIRK2^+/−^ mice) rescued the observed increase in GABA_B_-triggered currents in CA1 pyramidal neurons (Joshi et al., 2016). In agreement with triplication of KCNJ6 and the increase of GABA_B_/GIRK signaling, *V*_REST_ is hyperpolarized by 2.3 mV in CA1 pyramidal neurons and by 6.2 mV in DG granule cells (DGGC; Best et al., 2012; Kleschevnikov et al., 2012b). Of note, whereas no statistical difference was found in the hippocampus of Ts65Dn mice compared to WT for GABA_B_R subunit 1, GABA_B_R subunit 2 was slightly decreased, most likely due to a compensatory adaptation mechanism in response to the increase of GIRK2 levels (Best et al., 2012; Kleschevnikov et al., 2012b).

Apart from GABA_B_Rs, GIRKs channels may also mediate signaling from many other neurotransmitters including acetylcholine, dopamine, opioid, serotonin, somatostatin and adenosine through their metabotropic GPCRs (Lüscher et al., 1997). Therefore, GIRK2 triplication in DS may account for a large plethora of different effects depending on the neurotransmitter system involved. For example, GIRK2 channels also mediate the hypothermic response induced by administration of the serotonin 5-HT_1A_ and 5-HT_7_ receptor agonist 8-OH-DPAT (Costa et al., 2005). Indeed, 8-OH-DPAT hypothermic response is increased in Ts65Dn mice (Stasko et al., 2006).

### Synaptic Plasticity and GABA Signaling in DS

A large body of evidence linking altered GABA physiology to DS-related cognitive phenotypes came from the study of hippocampal synaptic plasticity in trisomic mice. LTP and LTD are widely accepted models of synaptic plasticity, characterized by the strengthening or weakening (respectively) of synaptic efficiency following specific stimulation protocols. In particular, LTP represents the cellular correlate of memory and it is necessary for different cognitive processes, including learning (Lynch, 2004; Whitlock et al., 2006; Nabavi et al., 2014). In line with the impairment in cognitive abilities characteristic of persons with DS and of DS mouse models, synaptic plasticity paradigms (both LTP and LTD) were found altered in Ts65Dn hippocampal slices (Siarey et al., 1997, 1999, 2006).

Given the already described pivotal role of GABAergic inhibition in regulating synaptic plasticity and given the defective GABAergic transmission in DS animals, researchers have evaluated the effect of GABAergic drugs on different forms of hippocampal LTP in DS mice. Interestingly, LTP occurring at Shaffer collateral-CA1 synapses (CA3-CA1 LTP) was rescued in Ts65Dn slices by acute application in the recording bath of the GABA_A_R antagonist picrotoxin (PTX). Similarly, potentiation at perforant path-DGGC synapses (DG-LTP) was restored by application of either PTX or the GABA_B_R antagonist CGP55845 (Kleschevnikov et al., 2004, 2012a; Costa and Grybko, 2005), establishing a causal link between GABAergic transmission and plasticity deficits in DS. Remarkably, CA3-CA1 LTP was also rescued by application of the NKCC1 inhibitor Bumetanide, indicating the involvement of depolarizing GABAergic signaling in the impairment of synaptic plasticity in DS (Deidda et al., 2015b). An even more striking evidence came about when it was shown that DG-LTP was rescued in acute slices from Ts65Dn mice that had been chronically treated with the GABA_A_R blocker pentylenetetrazole (PTZ). The effect was evident for up to 1–3 months after treatment cessation (Fernandez et al., 2007). Accordingly, chronic *in vivo* treatment with RO4938581, a selective negative allosteric modulator of α_5_-containing GABA_A_Rs, rescued *in vitro* CA3-CA1 LTP in Ts65Dn mice, although it was not assessed after drug withdrawal (Martínez-Cué et al., 2013).

Finally, comparative evaluation of synaptic plasticity deficits in the other DS mouse models has shown that DG-LTP was similarly impaired and rescued by PTX application in Ts1Cje, Ts1Rhr, and triple trisomic (Dp10/Dp16/Dp17) mice (Belichenko et al., 2007; Belichenko P. N. et al., 2009; Belichenko et al., 2015). Conversely, CA3-CA1 LTP was decreased in Ts1Cje, Dp16 and triple trisomic mice, unchanged in Ts1Rhr and Dp10 mice, and even significantly increased in Dp17 mice, but none of these later studies evaluated the possible contribution of GABAergic signaling (Siarey et al., 2005; Olson et al., 2007; Yu et al., 2010a,b).

Of note, beside the two studies performed in primary neuronal cultures (Best et al., 2007; Stern et al., 2015), all other electrophysiological investigations of GABAergic signaling alteration in DS mouse model have been performed on acute brain slices. Therefore, further studies are needed to assess whether GABAergic dysfunctions can be fully reproduced on *in vitro* neuronal cultures and, most importantly, if they are also occurring *in vivo* in the intact brain.

## Pharmacological Interventions Targeting GABA Transmission to Rescue Cognitive Deficits in DS Mouse Models

Given the large body of evidence showing the involvement of GABA signaling in neurophysiology, cognition and synaptic plasticity in DS, many studies have evaluated learning and memory processes in DS mice after pharmacological interventions targeting GABAergic transmission. Although therapy with GABA_A_R antagonists may be hampered by possible pro-epileptic and anxiogenic side effects in patients already at increased risk for these conditions, a first seminal study evaluated the efficacy of different GABA_A_R antagonists (PTX, PTZ and Bilobalide) at non-epileptic doses on long-term declarative memory in the novel object recognition test in Ts65Dn mice: chronic (but not acute) blockade of GABA_A_ transmission rescued object recognition memory after 2-weeks of treatment (Fernandez et al., 2007). Strikingly, the positive effect of PTZ (a drug previously used in the clinic) was maintained after an additional 2 months of drug withdrawal, indicating that the treatment likely induced long-term neuronal-circuit rearrangements able to sustain cognitive performance in trisomic mice. The positive effect of chronic PTZ treatment on learning and memory was further confirmed in the Morris water maze by a later study (Rueda et al., 2008). However, the same study showed a worsening effect of PTZ in Ts65Dn mice in a test for equilibrium, indicating a possible side effect of the drug. Interestingly, a follow-up study found that the effective dose of PTZ could be reduced by 10 times without compromising its efficacy, thus defining a potential safer therapeutic window with respect to the possible pro-epileptic and anxiogenic side effects of the drug (Colas et al., 2013).

Prompted by the effectiveness of PTZ treatment and by the observation that mice knock-out for the GABA_A_R α_5_ subunit—which is highly expressed in the hippocampus (Wisden et al., 1992)—show increased learning and memory performance (Collinson et al., 2002), a second series of studies has evaluated the efficacy of two inverse agonists selective for the α_5_ subunit of the GABA_A_R and acting as negative allosteric modulators. These two drugs had been developed by different pharmaceutical companies as cognitive enhancers: α_5IA_ (Chambers et al., 2003) and RO4938581 (Ballard et al., 2009). Both acute and chronic treatments with these drugs were proven effective in ameliorating cognitive performance of Ts65Dn mice in both the novel object recognition and Morris water maze tests without showing pro-epileptic or anxiogenic side effects that may be associated with GABA_A_R antagonism (Braudeau et al., 2011a,b; Martínez-Cué et al., 2013).

Moreover, following the observation that the chloride importer NKCC1 is upregulated in DS and that GABA_A_ transmission is depolarizing in adult Ts65Dn mice, we have recently assessed the efficacy of the NKCC1 inhibitor Bumetanide (a FDA-approved loop diuretic) on learning and memory in trisomic mice. We found that chronic NKCC1 inhibition in Ts65Dn mice was able to rescue discriminative memory in the object recognition test, spatial memory in the object location test and associative memory in the contextual fear conditioning task (Deidda et al., 2015b). Interestingly, the effect of Bumetanide was evident also after acute treatment and quickly lost upon treatment cessation, indicating that the effect of Bumetanide relied on direct NKCC1 inhibition rather than on neuronal-circuit rearrangements. These findings are in line with previous studies on the positive effect of GABA_A_R antagonists on learning and memory in DS mice. Indeed, GABA_A_R antagonists will reduce aberrant depolarizing GABA_A_ signaling in Ts65Dn mice regardless of whether GABA_A_R signaling is increased in DS. On the other hand, since modulation of [Cl^−^]_i_ is predicted to have only little effect on shunting inhibition, Bumetanide would preserve the ability of GABA_A_ currents to shunt concurrent excitatory inputs, hence possibly reducing potential pro-epileptic side effects. Conversely, GABA_A_R antagonists together with reducing receptor transmission will also reduce the shunting inhibition, thus possibly increasing the risk of seizures and profoundly altering neuronal input integration.

With respect to increased GABA_B_R signaling in DS, both acute and chronic treatment with the specific GABA_B_R antagonist CGP55845 restored cognitive performance in the novel object recognition test and associative memory in the contextual fear conditioning test in Ts65Dn mice (Kleschevnikov et al., 2012a), indicating also the possible involvement of metabotropic GABA signaling in DS cognitive impairment.

## GABA Signaling in DS Patients and Patient-Derived iPSC

Less detailed information is obviously available regarding the GABAergic system in individuals with DS. Reduced brain size and decreased density of neurons are hallmarks of DS and mainly arise from reduced neurogenesis during brain development (Colon, 1972; Sylvester, 1983; Wisniewski et al., 1984; Wisniewski and Schmidt-Sidor, 1989; Wisniewski, 1990; Kesslak et al., 1994; Wisniewski and Kida, 1994; Raz et al., 1995; Aylward et al., 1997, 1999; Teipel et al., 2003; Teipel and Hampel, 2006; Contestabile et al., 2007; Guidi et al., 2008). Nevertheless, only two histological studies on DS autoptic brain samples have selectively evaluated cell counts of *bona fide* GABAergic interneurons. One first study found decreased number of Golgi-stained aspinous stellate (putative GABAergic) cells in the somatosensory, visual and auditory cortices (Ross et al., 1984). Accordingly, the number of Parvalbumin and Calbindin-positive non-pyramidal neurons was also reduced in the frontal and temporal cortices of DS patients (Kobayashi et al., 1990). Although such decrease of GABAergic neurons may come from a secondary effect due to Alzheimer-like degeneration in DS, brain GABA concentration has been shown to be specifically decreased in AD, but not in aging DS patients (Seidl et al., 2001). Moreover, microarray studies to evaluate changes in gene expression on human DS cortical neuronal progenitor cells (hNPCs) in culture have shown gene changes indicative of decreased GABAergic interneuron genesis (Bhattacharyya et al., 2009). Moreover, the same study showed increased expression of the α_2_ subunit of the GABA_A_R_,_ and downregulation of the α_3_ and α_5_ subunits in trisomic cells compared to controls (Bhattacharyya et al., 2009). This GABA_A_R composition favoring the α_2_ over the α_5_ subunits may be indicative that GABA_A_R opening is fastened, possibly reducing the opportunity of shunting inhibition and generating quick PSPs, a condition that may be of particular relevance if GABA_A_-mediated transmission is depolarizing in humans. Indeed, we have shown that NKCC1 is overexpressed also in the brains of DS patients, establishing a direct parallel with the Ts65Dn model (Deidda et al., 2015b), and possibly suggesting depolarized *E*_Cl_ in humans as in animal models. Finally, GABA levels were found decreased or unchanged in neurochemical (Reynolds and Warner, 1988; Seidl et al., 2001; Whittle et al., 2007) and ^1^H MRS studies (Śmigielska-Kuzia and Sobaniec, 2007; Śmigielska-Kuzia et al., 2010) on human trisomic brains.

The recently developed technique for reprogramming somatic cells has opened the possibility of studying patient-derived neurons as a valuable tool for modeling neurological diseases (Takahashi et al., 2007; Park et al., 2008). This approach has been also applied to DS and has permitted the generation of different induced pluripotent stem cells (iPSCs) lines. In particular, two studies have used trisomic iPSCs to assess GABAergic neurogenesis and synaptogenesis upon induction of neuronal differentiation. The results from these studies have highlighted a general impairment in synaptogenesis of DS iPSC-derived neurons that was mirrored by a decrease in the frequency of both inhibitory and excitatory spontaneous postsynaptic currents (sPSCs). However, the percentage of neurons expressing GABAergic markers, the fraction of GABAergic synapses, or the ratio of glutamatergic to GABAergic sPSCs were substantially unaffected (Weick et al., 2013; Hibaoui et al., 2014).

Overall, although the available data from human studies—while limited—seem not to support the data derived from animal research of increased GABA-mediated transmission due to the overproduction of GABAergic interneurons, further electrophysiological studies on iPSCs-derived neurons are needed. On the other hand, no study has yet assessed the occurrence of depolarizing GABA_A_ signaling or increased GABA_B_-mediated transmission in human DS neurons.

## GABAergic Drugs in DS Clinical Trials

Due to the encouraging preclinical data highlighting a strong link between GABA signaling and DS cognitive impairment, some of the pharmacological interventions effective in DS mouse models have been translated into clinical trials on individuals with DS. In particular, although the use of PTZ on DS patients has been questioned due to the potential pro-epileptic side effects, the dosage effective in rescuing learning and memory in Ts65Dn mice is well below the epileptic dose (Colas et al., 2013). Indeed, Balance Therapeutics is conducting a clinical trial in Australia to evaluate the efficacy of PTZ (BTD-001) on individuals with DS. This COMPOSE trial (Cognition and Memory in People with DS, registered in the Australian-New Zealand clinical trial registry as ACTRN12612000652875) is a phase IB study aimed at evaluating safety, tolerability, preliminary efficacy and pharmacodynamics of BTD-001 at two different doses in adults and adolescents with DS. Recruitment for this study has been completed^2^ and the results are eagerly awaited.

Moreover, Hoffmann-La Roche has conducted two clinical trials for assessing the efficacy of the selective negative allosteric modulator of the α_5_-containig GABA_A_R Basmisanil (RG1662/RO5186582), a derivative of RO4938581, previously shown to rescue learning and memory in Ts65Dn mice (Martínez-Cué et al., 2013). The first trial (CLEMATIS) was designed as a phase II placebo-controlled study (NCT02024789) aimed at evaluating the efficacy and safety of RG1662 at two different doses in adults and adolescents with DS. Disappointingly, although the complete results of the CLEMATIS trial have not been disclosed yet, a media release from Roche later this June has announced that the study did not meet its primary and secondary endpoints on improving cognitive functions in DS patients, and that there was no significant difference between the placebo and the treated groups^3^. The lack of efficacy seen in the CLEMATIS trial induced the discontinuation of the second ongoing placebo-controlled dose-investigating pediatric study (NCT02484703), aimed at evaluating pharmacokinetics, pharmacodynamics, efficacy, and safety of RG1662 in children with DS. The interruption was not decided for safety reasons, as the drug appeared to be well-tolerated and no relevant side effects were observed.

## Speculation for Future Directions in The Research on DS and GABAergic Transmission

### Possible Convergence of Different Drug Treatments on GABAergic Signaling in DS

Alternative mechanisms, apart from GABA signaling, have been shown to underline LTP and/or cognitive deficits in Ts65Dn mice. Indeed, CA3-CA1 LTP and/or behavioral performances were rescued in Ts65Dn mice by a variety of manipulations including: acute application of the GluN2B-selective antagonist Ro25–6981 (Hanson et al., 2013) or of the green tea polyphenolic compound epigallocatechin-3-gallate (EGCG, an inhibitor of the DS triplicated kinase Dyrk1A; Xie et al., 2008), chronic treatments with polyphenolic green tea extracts enriched in EGCG (De la Torre et al., 2014; Catuara-Solarz et al., 2015), the monoacylglycerol lipase inhibitor JZL184 (Lysenko et al., 2014), the neuro-hormone Melatonin (Corrales et al., 2013), the Sonic Hedgehog agonist SAG1.1 (Das et al., 2013), the serotonin reuptake inhibitor Fluoxetine (Bianchi et al., 2010b; Begenisic et al., 2014; Guidi et al., 2014), or also exposure to an enriched environment (EE; Begenisic et al., 2011, 2015). Nevertheless, most of these treatments are also known to directly or indirectly modulate the GABAergic system, while for others such link has not been established yet. For example, Ro25–6981 selectively reduced the activation of GABAergic interneurons in the Stratum Radiatum of the hippocampus (Hanson et al., 2013), whereas JZL184 decreased GABAergic transmission by likely modulating presynaptic cannabinoid receptors (Katona et al., 1999; Zhang et al., 2009; Lee et al., 2015). Instead, both treatment with Fluoxetine and exposure of Ts65Dn mice to EE showed beneficial effects on LTP and memory in Ts65Dn mice, possibly by reducing release from GABAergic terminals (Begenisic et al., 2011, 2014, 2015). Indeed, Fluoxetine has been previously found to reduce GABAergic neurotransmission in the hippocampus independently from the inhibition of serotonin reuptake (Méndez et al., 2012; Caiati and Cherubini, 2013), and to decrease the levels of extracellular GABA *in vivo* (Maya Vetencourt et al., 2008). Additionally, Fluoxetine may also inhibit GIRK channels (Kobayashi et al., 2003; Cornelisse et al., 2007), therefore possibly normalizing enhanced GABA_B_R signaling in Ts65Dn mice. In this regard, it is important to note that GIRK channels are also coupled to serotonin receptors 5-HT_1A_ (Williams et al., 1988; Llamosas et al., 2015; Montalbano et al., 2015) and GIRK2 triplication can therefore impact on serotoninergic signaling in DS. Indeed, stimulation of 5-HT_1A_ receptors in the hippocampus can reduce neuronal firing frequency and gamma oscillations through GIRK channels activation (Johnston et al., 2014). Since fluoxetine treatment reduces GIRK-mediated 5-HT_1A_ and GABA_B_ receptor signaling in the dorsal Raphe (Cornelisse et al., 2007), this mechanism may be involved in the therapeutic effect of fluoxetine in Ts65Dn mice. However, a possible link between changes in serotonin signaling due to GIRK2 overexpression and the effects of fluoxetine on learning and memory has not been assessed in DS mice. On the other hand, chronic treatment with polyphenolic green tea extracts enriched in EGCG decreased the expression of the GABAergic synaptic markers GAD67 and VGAT in the cortex of Ts65Dn mice (Souchet et al., 2015). Conversely, EGCG positively modulated GABA_A_-mediated transmission when administered acutely (Vignes et al., 2006; Park et al., 2011). Of note, a recently concluded clinical trial (TESDAD, NCT01699711) evaluating the efficacy of long-term green tea extract treatment on DS patients showed some behavioral improvements, although—as stated by the authors—below the threshold for clinical relevance in 2 out of 15 measured tests (de la Torre et al., 2016). Finally, the positive effect of Melatonin is unlikely to depend on decreased GABAergic signaling because it acts as a *positive* allosteric modulator of GABA_A_ receptors (Wang et al., 2003; Scott et al., 2010; Cheng et al., 2012). Altogether, these data indicate that a number of the large plethora of effective drug treatments able to restore LTP and/or memory in Ts65Dn mice may rely on a common ground of action through the modulation of the GABAergic system.

### Excitatory Deficits in DS

Altogether, the literature reported above indicates a key role for GABAergic signaling in neuronal network deficits in trisomic mice. However, deficits in excitatory inputs and glutamatergic transmission could also contribute to the imbalance in excitatory/inhibitory transmission in the trisomic brain. Indeed, several lines of evidence indicate delayed development and decreased production of excitatory glutamatergic neurons in the cortex (Chakrabarti et al., 2007, 2010; Tyler and Haydar, 2013; Guidi et al., 2014), DG (Lorenzi and Reeves, 2006; Contestabile et al., 2007; Bianchi et al., 2010b) and cerebellum (Baxter et al., 2000; Roper et al., 2006; Contestabile et al., 2009) of Ts65Dn mice. Accordingly, a decreased density of glutamatergic synapses was found in the cortex and hippocampus of Ts65Dn mice by electron microscopy and immunohistochemistry (Kurt et al., 2000, 2004; Chakrabarti et al., 2007; Rueda et al., 2010; Guidi et al., 2013; Stagni et al., 2013; García-Cerro et al., 2014), as well as in human DS iPSCs-derived neurons (Weick et al., 2013; Hibaoui et al., 2014). However, *in vivo* ^1^H MRS evaluation of different metabolites and neurotransmitters in the Ts65Dn hippocampus showed no difference in the concentration of either GABA or glutamate (Santin et al., 2015). Despite of the evidence supporting a possible decrease of excitatory inputs, few electrophysiological studies have functionally evaluated glutamatergic signaling in trisomic mice. Decreased frequency of miniature excitatory postsynaptic currents (mEPSC) was found in the CA3 hippocampal region (Hanson et al., 2007; Stagni et al., 2013), whereas a decreased ratio of postsynaptic NMDA/AMPA-evoked responses was found in the CA1 region of Ts65Dn mice (Das et al., 2013). Interestingly, Ts65Dn mice show increased electrophysiological and behavioral response to pharmacological manipulations of NMDA receptors (Costa et al., 2007; Scott-McKean and Costa, 2011). Although a complete mechanistic explanation behind such effect will need further investigations (Costa, 2014), inhibition of NMDA transmission with the noncompetitive antagonist Memantine rescued learning and memory performance in different behavioral tests both after acute and chronic administration in Ts65Dn mice (Costa et al., 2007; Rueda et al., 2010; Lockrow et al., 2011). Following these studies, two clinical trials evaluated the efficacy of Memantine on improving cognitive functions in DS patients. Although the drug was well-tolerated, the results of the first study (NCT00240760) did not show any improvement on cognitive functions (Hanney et al., 2012). However, a pitfall of this study may be represented by the advanced age of participants. Indeed, since DS patients are at increased risk of developing Alzheimer degeneration early in life, irreversible pathological and/or degenerative changes may have been already in place by the time of treatment (Costa, 2014). A second trial (NCT01112683) on a relatively small number of young-adults with DS showed no significant difference in the primary outcome. However, some encouraging improvements were detected in a secondary measure of verbal memory (Boada et al., 2012). Overall, the lack of a comprehensive assessment of glutamatergic functions in DS mouse models will require a more detailed investigation to clearly evaluate the involvement of glutamatergic signaling in excitatory/inhibitory imbalance in the trisomic brain.

### GABA in Neurodevelopment and Critical Period Plasticity in DS

DS is widely recognized as a neurodevelopmental disorder since many (but not all) brain deficits originate during the embryonic and early life. Since activation of both GABA_A_R and GABA_B_R plays a key role in brain development (Gaiarsa and Porcher, 2013; Le Magueresse and Monyer, 2013), changes in ambient GABA (also due to increased number of GABAergic interneurons) may underline at least some of the brain alterations that originate during DS fetal life and persist into adulthood. Nevertheless, no study has so far addressed the role of aberrant GABAergic signaling in neural circuit formation in DS. In particular, it would be interesting to evaluate the timing of the depolarizing/hyperpolarizing GABA switch (Ben-Ari, 2002) and related developmental changes in GABA_A_R-subunit expression (Succol et al., 2012) in trisomic mice. On the other hand, GABA_B_R signaling may affect DS brain development by modulating adenylate cyclase and calcium channels. Triplication of GIRK2 is instead unlikely to directly contribute to GABA_B_-mediated early developmental DS brain alterations, as coupling of GABA_B_Rs with GIRK channels does not occur until the second postnatal week of life in rats (Fukuda et al., 1993; López-Bendito et al., 2003; Bony et al., 2013). Nevertheless, the possibility of premature coupling of GABA_B_Rs to GIRK2 due its overexpression in DS may still exist.

Anyhow, future studies are also needed to investigate whether modulating GABAergic signaling and/or intracellular Cl^−^ accumulation during specific developmental periods, when brain circuits are possibly more prone to plastic changes, may result in beneficial effects in learning and memory that persist into adulthood. In this regard, it would be interesting to test the long-term effects of GABA_A_R inhibition (i.e., PTZ or α5 negative allosteric modulator treatments) or of lowering [Cl^−^]_i_ with Bumetanide during brain development. The importance of an early intervention during a likely essential period of brain development in DS is highlighted by the observation that different treatments (e.g., Fluoxetine, SAG1.1 and Choline) administered during gestation or in the early postnatal period rescued memory performances in Ts65Dn mice later in life (Bianchi et al., 2010b; Moon et al., 2010; Das et al., 2013; Guidi et al., 2013; Velazquez et al., 2013; Ash et al., 2014). Nevertheless, one important and apparently neglected aspect of early pharmacological interventions is related to possible side effects of drug treatment during development, which should in fact be carefully considered. Indeed, similarly to the beneficial effect of the drugs, adverse effects may persist into adulthood and may impact on the functionality of different organs or systems. A recent example came from the NEMO trail (NCT01434225) regarding the use of Bumetanide on newborns with hypoxic ischemic encephalopathy. Indeed, the trial was interrupted because of hearing loss adverse effect (Pressler et al., 2015). Moreover, in light of the encouraging results obtained on Ts65Dn offsprings prenatally and perinatally treated with Fluoxetine (Bianchi et al., 2010b; Guidi et al., 2014), a clinical trial has been recently announced^4^, aimed at assessing the efficacy of Fluoxetine administration during the prenatal (2nd trimester) and postnatal (up to 2 years of age) periods in ameliorating the developmental abilities of children with DS. However, the dosage and timing of administration should be carefully considered in the light of the fact that Fluoxetine administration during pregnancy has been associated to birth defects (Reefhuis et al., 2015), and treatment in adult patients increased seizure susceptibility (Pisani et al., 1999). Moreover, Fluoxetine treatment in rodents during embryonic and early postnatal life modifies the migration of cortical GABAergic interneurons, and—later in life—increases aggression in males as well as changes emotional and social behaviors (Kiryanova et al., 2013; Ko et al., 2014; Frazer et al., 2015; Svirsky et al., 2016).

Aberrant GABAergic transmission may also affect the critical-period plasticity in the visual cortex. Interestingly, this critical-period plasticity depends on the depolarizing action of GABA during early development and its length can be extended by reducing depolarizing GABA_A_ signaling by treatment with Bumetanide (Deidda et al., 2015a). Therefore, an early intervention with Bumetanide may provide an extended window for neuronal plasticity in DS. Although no study has assessed critical-period plasticity in visual cortical circuits of trisomic animals, Ts65Dn mice show deficits in cortical visual evoked potentials (VEPs; Scott-McKean et al., 2010). Interestingly, exposure to EE either during development or adulthood restored cortical VEP responses in Ts65Dn mice, possibly through modulation of GABAergic transmission (Begenisic et al., 2011, 2015).

### GABA in Other DS Symptoms, Possibly Affecting Cognition

Besides cognitive impairments, individuals with DS present a number of other symptoms (i.e., epilepsy, sleep disorders and anxiety) that affect the quality of their lives and may in turn also impinge on their cognitive abilities (Pueschel et al., 1991; Stafstrom et al., 1991; Haw et al., 1996; Carter et al., 2009; Lott and Dierssen, 2010; Breslin et al., 2011; Rissman and Mobley, 2011; Vicari et al., 2013; Angriman et al., 2015; Edgin et al., 2015; Robertson et al., 2015; Konstantinopoulou et al., 2016; Maris et al., 2016). Interestingly, epilepsy, sleep disorders and anxiety have all been associated to defective GABAergic transmission (Wagner et al., 1997; Choi et al., 2008; Rudolph and Knoflach, 2011; Möhler, 2012; Kaila et al., 2014b).

DS patients and Ts65Dn mice demonstrate increased incidence of epileptic seizures (Stafstrom et al., 1991; Westmark et al., 2010; Rissman and Mobley, 2011; Lott, 2012; Robertson et al., 2015). These observations appeared contradictory when considering that increased GABA signaling in Ts65Dn mice was expected to overall decrease neuronal network activity, and therefore reduce incidence of seizures. However, the increased incidence of seizures is in line with depolarizing GABA_A_ signaling in DS, because the shift in GABA_A_R-mediated responses will also clearly impact on the excitatory/inhibitory balance and promote brain neuronal circuit hyperexcitability. Interestingly, administration of γ-butyrolactone (GBL, a prodrug for the GABA_B_R agonist γ-hydroxybutyrate: GHB) induced epileptiform activity in Ts65Dn mice that was rescued by genetically restoring GIRK2 gene dosage to disomy (Cortez et al., 2009; Joshi et al., 2016). Although the general molecular mechanism of GHB action is still matter of debate (Bay et al., 2014; Venzi et al., 2015), it is intriguing to speculate that, in the scenario of GABA dysregulation in DS, both the GABA_B_R-GIRK2 signaling and depolarizing GABA_A_ signaling may play a role in GBL-induced epileptic phenotype seen in Ts65Dn mice. Indeed, GHB would further increase the already enhanced GIRK2-mediated signaling, thus abnormally drifting *V*_REST_ towards hyperpolarization. This condition would emphasize the depolarizing GABA_A_ signaling, with amplification of all depolarizing inputs and consequent trigger of epileptic activity. On the other hand, Ts65Dn mice also show increased incidence of audiogenic seizure that can be reduced by treatment with the metabotropic glutamate receptor subtype mGluR_5_ antagonist Fenobam (Westmark et al., 2010), but not by inhibition of NKCC1 with Bumetanide (Deidda et al., 2015b). Moreover, also the characteristic hyperactivity of Ts65Dn mice (Escorihuela et al., 1995; Reeves et al., 1995; Sago et al., 2000) was reduced by the α_5_-containing GABA_A_R negative modulator RO4938581 (Martínez-Cué et al., 2013), but not by Bumetanide treatment (Deidda et al., 2015b). These observations indicate that more complex mechanisms in addition to altered GABA signaling may underline these increased seizure susceptibility and hyperactive phenotypes in Ts65Dn mice.

DS patients have also higher incidence of sleep disturbance in relation to the general population (Carter et al., 2009; Breslin et al., 2011; Edgin et al., 2015; Konstantinopoulou et al., 2016; Maris et al., 2016), and Ts65Dn mice show some sleep alterations mainly consisting in increased awaking and higher theta power in sleep EEG (Colas et al., 2008). On the other hand, Ts65Dn mice show little or no differences in circadian rhythms, which are not altered by PTZ treatment (Stewart et al., 2007; Ruby et al., 2010). Nevertheless, it is striking the observation that PTZ was effective in restoring memory performances in Ts65Dn mice only when it was administrated during the light phase of the day, but not during the dark phase, indicating a possible different circadian contribution of the GABAergic system on learning and memory in DS (Colas et al., 2013). Given the recently identified connection between GABA signaling and memory in circadian arrhythmic animals (Ruby et al., 2008, 2013), more studies are needed to uncover this possible relationship in DS. Since GABA_A_ signaling (and possibly Cl^−^ homeostasis) has been repeatedly described as a key regulator of sleep and circadian rhythms (Wagner et al., 1997; Choi et al., 2008), it is possible that alterations of GABA signaling and/or expression of NKCC1 may play a role in sleep disorders in DS.

Finally, the strong involvement of GABA_A_ signaling in anxiety disorders (Rudolph and Knoflach, 2011; Möhler, 2012) and the observation that DS patients show increased anxiety (Vicari et al., 2013) may indicate that also this aspect possibly originates, at least in part, from the alteration of GABA_A_ signaling. However, this issue has not been addressed yet in DS mice. On the other hand, since GIRK2 knockout mice show reduced anxiety-related behavior (Pravetoni and Wickman, 2008) but GABA_B_R knockout mice show an anxious phenotype (Mombereau et al., 2005), coupling of GIRK channels to metabotropic neurotransmitter receptor systems other than GABA_B_Rs may play a role in regulating anxiety-related behaviors in DS. For example the overexpression of GIRK2 in the midbrain of Ts65Dn mice (Harashima et al., 2006) may modulate signal transduction of dopamine receptors (Perez et al., 2006; Podda et al., 2010; Marcott et al., 2014; Zhao et al., 2016) and impact on different DS phenotypes including anxiety-related symptoms (Sim et al., 2013). Finally, a possible role for altered GABAergic transmission in impaired adult neurogenesis (Song et al., 2012; Pallotto and Deprez, 2014) and AD (Li et al., 2016)-which both eventually result in impaired cognition- is still an unexplored field of research in DS (Rissman and Mobley, 2011).

## Concluding Remarks

The genetic cause of DS has been unequivocally identified in the triplication of genes located on the human chromosome 21, although the exact group of genes and the pathological mechanisms underlying DS intellectual disability are still unclear. Despite of the limitations in reproducing in mice the exact genetic condition characterizing DS human pathology, the creation of DS mouse models with construct and face validity has been giving nevertheless a wide contribution in understanding gene-phenotype association, DS pathological processes, and in extending therapeutic prospects. Here, we evaluated the evidence pointing at a role for abnormal signaling from GABA_A_ and GABA_B_ receptors in the neuronal defects associated with DS, and we considered particularly the Ts65Dn mouse model of DS, one of the most largely used. The origin of defective GABAergic transmission tracks back into early neurodevelopment, with possible excitatory/inhibitory unbalance and neuronal and plasticity impairments that persist into adulthood. However, the weight of the GABAergic inhibitory vs. the glutamatergic excitatory transmission in this unbalance is still unclear. Indeed, depolarizing GABA_A_ signaling coexists with glutamatergic excitatory alterations in Ts65Dn mice, and excitatory/inhibitory unbalance appears to be brain-region specific in DS mouse models. Moreover, possible decreased GABAergic transmission in human DS patients may seem to be discordant with the increased GABAergic transmission in Ts65Dn mice, although to date, human data are still scarce and inconclusive. Fortunately, the recent advances in the use of human-derived iPSCs may give a large contribution in the future understanding of DS neuropathology, with higher translational viability. Indeed, whether the failure of the CLEMATIS trial and limited positive results from other clinical studies can be ascribed to inefficacy of the drugs (despite the strong preclinical data), a low predictive potential of the Ts65Dn mouse model may also have played a role. For example, changes in GABA_A_R subunit composition specific for human DS (Bhattacharyya et al., 2009), but lacking in Ts65Dn mice, could account for the failure of pharmacological treatments with a α_5_ selective antagonist in individuals with DS. Since the “perfect” mouse model does not exist yet, it will be worth for the future to assess the efficacy of each new pharmacological treatment in more than one DS mouse model, and promote parallel human and animal studies. On the other hand, the failures of clinical trials may also rest in the inadequacy of current neuropsychological tests in measuring cognitive improvements in DS (Gardiner, 2010, 2014; Fernandez and Edgin, 2016). Indeed, cognitive performance measured by tests are mostly dependent by the integration of different cognitive domains, thus the selective improvement of one of them may not be detected; on the other hand, an improvement in neuropsychological tests may not imply a perceived substantial improvement in the daily life.

In conclusion, the data reported above clearly highlight the multifaceted nature of DS brain abnormalities. These alterations represent the sum of different molecular mechanisms that most likely include impaired GABAergic transmission. Possibly, these abnormalities originate (at least in part) during development and lead to complex synaptic, physiological and circuit changes, ultimately causing cognitive deficits and other neurological manifestations. As a consequence, the still unmet need of identifying effective pharmacological interventions to alleviate DS-related cognitive impairment represents an incredible complex challenge for the future. Surely, the insights about GABA-related impairments in DS models may be of great relevance also for other neurodevelopmental disorders where defective GABAergic transmission (including depolarizing GABA_A_ signaling) may play a pathological role (e.g., Autism, Fragile X syndrome, epilepsy, and possibly Rett syndrome; Cellot and Cherubini, 2014; Deidda et al., 2014; He et al., 2014; Khazipov et al., 2015).

## Author Contributions

AC, SM and LC contributed to the conception and writing of the manuscript.

## Funding

This work was supported by Jérôme Lejeune Foundation (grants 254-CA2014A to AC, and 1266_CL2014A to LC) and Telethon Foundation (grants GGP15043 to AC and GGP13187, TCP15021 to LC).

## Conflict of Interest Statement

AC and LC are named as co-inventors on International Patent Application PCT/EP2014/078561, filed on December 18, 2014, and connected US, EP, JP National Phase Applications, claiming priority to US Provisional Application US 61/919,195, priority date December 20, 2013. The other author SM declares that the research was conducted in the absence of any commercial or financial relationships that could be construed as a potential conflict of interest.

## Footnotes

^1^http://vega.sanger.ac.uk

^2^http://www.anzctr.org.au

^3^http://www.roche.com/media/store/statements.htm

^4^http://www.utsouthwestern.edu/research/fact/detail.html?studyid=STU%20032014-006
